# Employees and the National Insurance Act

**Published:** 1912-07-13

**Authors:** 


### NATIONAL INSURANCE ACT DEPARTMENT.

### EMPLOYEES AND THE NATIONAL INSURANCE ACT.

### XIX.—Insurance Cards and Books.

The National Insurance Act will have commenced its operation before our next issue, and millions of employed persons and their employers will have felt its first effect by the payment of the first weekly contribution. The past fortnight has been one of unprecedented activity at the Offices of the Insurance Commissioners and with the Approved Societies. A stupendous task has had to be performed in supplying the insured persons with the necessary Insurance Cards and Books, and the Post Offices throughout the United Kingdom have been inundated with applications for cards. The vast majority of persons affected by the Act had continued to hope that the Act would be deferred in its operation, in spite of all the Government assurances to the contrary. In consequence of this the applications for membership of approved societies were deferred as long as possible, with the result that it became an almost impossible task to cope with the rush during the last few days. No card has been furnished to an insured person except on the receipt of a written application, and every card has had to be filled in by the issuer with the name and address of the person to whom it has been issued. It can at once be seen that the amount of clerical work that has been entailed has been enormous, and those who have had to wait for their cards longer than they had expected will, doubtless, appreciate the difficulties that have beset the authorities during the past fortnight.

Insurance Books Described.

Besides the Insurance Cards, to which the stamps have to be affixed, every insured person will be furnished wih an Insurance Book. The purpose of this book is to record the contributions paid and the benefits received by the insured person. Entries in the book must not be made by the insured person, but only by an official of the society of which the person is a member. The book corresponds in all respects to the pass books issued by banks, but furnishes in addition an amount of printed matter sufficient to enable the holder to learn all that is necessary in regard to the rates of contribution and benefit applicable to his own circumstances. Unlike the contribution card, the Insurance Book plainly sets forth the name of the society to which the insured person belongs, and the number given him by the society for purpose of reference. It will, doubtless, be remembered that it is the intention of the Act that the employer shall not know the particular society to which his employee belongs. Hence it is clearly laid down that the name of the society must not be shown on the Insurance Card until the expiration of the period of its currency. The employer has no right to require the production of the Insurance Book, and, in consequence, there is no objection to the name of the society appearing thereon. In fact, it is necessary that the name of the society and the member's number should appear in order that the contributions and benefits shall be properly recorded in the books of the particular society.

There are many varieties of cards and books. The two principal classes are those which refer to employed contributors and employed persons aged sixty-five and upwards; the cards for men are of a grey colour and those for women are of a buff colour. The colours of the stiff covers of the Insurance Books correspond to the colours of the cards. There are other varieties for aliens, persons employed in the mercantile marine service, voluntary contributors, and others. In course of time we shall become acquainted with the forms and books by force of habit, but it is distinctly bewildering to the ordinary person at the outset to grasp all the matters which necessitate this differentiation of class from class, and the attendant varieties of cards and books.

Hard Cases—Seasonal Employment.

The initiation of a scheme of such magnitude as that of the National Insurance Act is bound to reveal cases of apparent hardship, and many curious anomalies have been brought to our notice. There is, for instance, the case of those who are only in regular employment for a comparatively short period each year. We are writing at a fashionable seaside resort where we learn that some hundreds of the normal population are only regularly employed for a comparatively short season—some three or four months each year. The special provisions of the Act as to seasonal employment do not here apply as the persons to whom we refer are not employed throughout the year. In the case of seasonal trades where systematically there are periods of inflation and depression of work, it is possible for an arrangement to be entered into with the Insurance Commissioners whereby the rate of contributions payable during the period of short work is at a lower scale than the normal as prescribed in the Act, while a corresponding addition is made to the rate when work is heavy. This can only apply to such persons who are permanently employed throughout the year, and the case to which we refer, of those who find employment as domestic servants, waiters, and the like during a short summer season at a seaside resort and then return without apparent means of subsistence to private life for the rest of the year, does not seem to be covered by the special provisions. Whilst such persons are at work it would seem that they must be insured, that is to say, their contributions will have to be paid, unless, of course, they can obtain special exemption on the ground either that they have a pension or other unearned income of at least £26 a year, or that they are mainly dependent upon some other person for their livelihood. Even when so exempted the employer's contribution is payable and no benefit is directly received by the employee from such payment. In hundreds of cases no such exemption will be able to be claimed, and in these cases the position will apparently be one in which the insured will always be in arrear to such an extent that it will not be possible to claim benefit unless the insured person pays the full contribution —the employer's as well as his own—whilst he is off work. The payment of 7d. a week in the case of men, and of 6d. a week in the case of women, when no income is being received is not a pleasant prospect, and the accumulated charge to be made good during the period whilst the insured is at work, if he elects to allow the arrears to accumulate, will amount to a substantial sum.

### Lives nearly Seventy on Entry into Insurance.

Another case of seeming hardship is that of an employed person who is at the present time very nearly seventy years of age. Contributions are payable until the attainment of the age of seventy, and according to the Act the sickness and disablement benefits cease at that age. There is, however, a waiting period of six months during which no benefit can be received. To cite an example—suppose on July 15 an employed person is aged exactly sixty-nine years and six months; contributions will have to be paid by his employer on his behalf for the six months until he attains the age of seventy, at which age the benefits nominally cease, but during the six months which exactly corresponds with the waiting period the insured person has not been entitled to benefit. Thus it would seem that six months' payments have to be made without the possibility of obtaining any benefit therefrom.

Fortunately this case has received attention, and in a scheme approved by the Commissioners, that may be adopted by any approved society for its aged members, it is laid down that, after the expiration of the waiting period, those persons who at the time of entry into insurance are over sixty-nine years of age shall be entitled if they are ill to as many weeks' sickness benefit, at the reduced rate prescribed, as they have paid weekly contributions, even though the period as thus modified to which the payment of benefit applies extends beyond the age of seventy.

This is a fairly satisfactory solution of the difficulty, but it should be noted particularly that because a person has paid, say, 26 weeks' contributions he does not necessarily actually receive 26 weeks' sickness benefit. He is only entitled to that number of weeks' sickness payments should he actually be ill and incapable of work.

### How Approved Societies Get Their Money.

We have found that a large number of people are unable to understand how the approved societies will obtain the funds out of which to pay the benefits. The process is rather roundabout, but very effective nevertheless. In the first place the money is paid into the Post Office when the employer obtains his supply of stamps. As the stamps are special National Health Insurance Stamps and not ordinary postage stamps the Post Office will know, simply as a matter of accounting, from the number of stamps sold how much money must be credited to the Insurance Commissioners. The stamps so purchased are then affixed to the Insurance Cards and these are forwarded at the end of each quarter by the insured persons to the societies to which they belong. After the cards have been examined by the society and the amounts represented by the stamps have been credited in the books of the society to the individual accounts of its members, they will be sent for re-examination to the Insurance Commissioners, with a claim for the total sum represented by the stamps affixed thereto. As soon as the Commissioners are satisfied as to the validity of that claim the amount will be credited to the funds of the particular society, and thus the amount originally paid by the employer across the Post Office counter will arrive at its final destination, and there be ready for use as occasion demands to pay the benefits as they accrue. The societies will not, therefore, be in possession of funds until some time during the second quarter, and this is one of the reasons for the institution of the waiting period of six months before the benefits commence.

### Answers to Correspondents.

### EXEMPTION CERTIFICATES.

Bala.—If you claim exemption from insurance on the ground that you have an unearned income of at least £26 a year, you will not have to pay the contribution applicable to an employee. Your employer will, however, be required to pay the employer's contribution of 3d. a week. This provision was inserted in the Act so that there should not be competition amongst employers for the employment of exempted persons. As you will personally not be paying anything, you will not be entitled to receive anything. The money derived from the employer's contributions will be devoted to the general insurance fund, and will, presumably, be used in the provision of sanatorium benefits and possibly also towards liquidating the initial deficiency under which the scheme is launched. It should be noted that at the commencement of the Act only those persons are entitled to become voluntary contributors who are regularly engaged in some occupation on their own account, and not as employed persons, and whose income from all sources does not exceed £160 a year. If you are employed within the meaning of that term as defined in the Act, you are not entitled to become a voluntary contributor, but you must become an insured person, unless you obtain a certificate of exemption on the grounds above mentioned.

### A TRAINED NURSE WORKING ON HER OWN ACCOUNT.

Nurse E. R., Boldon.—As you are working on your own account you do not come within the compulsory provision of the Act. If your age is not more than sixty-five, and you are regularly engaged in your occupation, you may join as a voluntary contributor, provided your total income from all sources does not exceed £160 per annum. The rate of contribution will depend upon your age at entry into insurance, and will be on a higher scale if you do not enter before January next. The Nurses' Insurance Society should be chosen if you decide to avail yourself of National Insurance.

NOTE.—Questions and Correspondence on all doubtful points are invited, and should be addressed to the Editor, The Hospital, 28 Southampton Street, Strand, W.C., and marked "Insurance."

